# Immunological, anti-inflammatory, and anti-oxidant effects of Fig and Olive leaves extracts nanoparticles against *Schistosoma mansoni* in C57BL/6 mice

**DOI:** 10.1007/s10787-026-02243-0

**Published:** 2026-05-20

**Authors:** Naira A. El-Attar, Eman A. El-Shabasy, Mamdouh R. El-Sawi

**Affiliations:** Zoology Department, Faculty of Science, Mansoura University, Mansoura, Egypt

**Keywords:** Schistosomiasis, Nanoparticles, Drug development, Biomarkers, Comet assay

## Abstract

Schistosomiasis, caused by worms of the genus *Schistosoma*, especially *S. mansoni*, remains a major global health challenge, with over 200 million people infected annually. Notably, sterile immunity does not naturally develop, and drug selection pressure has intensified due to the extensive use of praziquantel (PZQ) in endemic areas for nearly four decades, in addition to the emergence of resistance and lack of sterile immunity. This study investigated the effects of nanoparticle-loaded with natural plant extracts-*Ficus carica* (Fig) and *Olea europaea* (Olive)-administered in vivo at all *S. mansoni* developmental stages: cercariae (G4), schistosomulae (G5), immature worms (G6), and mature worms (G7). The primary objectives were to determine which stage was most susceptible to be eliminated in infected C57BL/6 mice. Fig and Olive were used in this study based on the oath in the wholly Quran. The results demonstrated a progressive improvement from G4 (Lowest) through G7 (Best) in immunological parameters, oxidative stress markers, inflammatory and apoptotic profiles, comet assay results, and liver function tests.

**Graphical abstract:**

**Figure:**
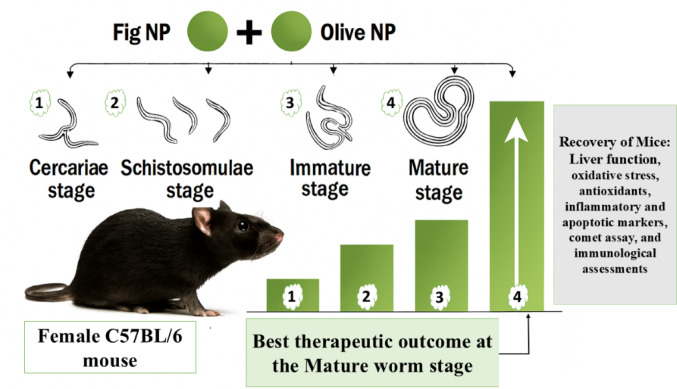
Effects of Fig and Olive nanocapsules on different *S. mansoni* stages (Fig. 1)

**Supplementary Information:**

The online version contains supplementary material available at 10.1007/s10787-026-02243-0.

## Introduction

Schistosomiasis is one of the most prevalent neglected tropical diseases, caused by parasitic trematodes of the genus *Schistosoma.* In 2022, 264.3 million people were recorded as needing treatment for schistosomiasis. The majority of cases in Africa are caused by infection with *S. mansoni* or *S. haematobium*. Every year, 200,000 people die from severe illness (Houlder et al. 2025).

The morbidity caused by *S. mansoni* infection is based on: the density of eggs trapped in tissues, which tissue is affected, the infection intensity, and the immune response and inflammation to these eggs. The types of schistosomiasis mansoni are acute and chronic. Acute disease can manifest as diarrhea and/or abdominal pain, while chronic disease typically pertains to a complicated immune-mediated disease associated with the presence of parasite eggs in the liver, gut, and surrounding tissues, resulting in fibrosis and, in advanced cases, organ failure (Pirzamana et al. 2024).

**Fig. 1:**
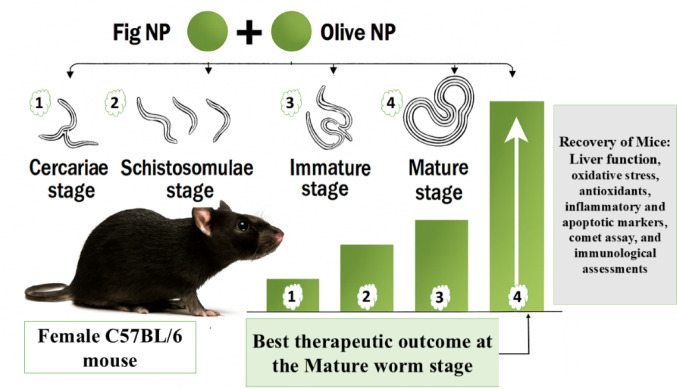
Effects of Fig and Olive nanocapsules on different *S. mansoni* stages

Female C57BL/6 mice are typically utilized as hosts in severe diseases because of their robust immunity, which protects them from the majority of complement-driven ailments, including sepsis, bilharzia, ischemia/reperfusion injury, and graft rejection. It’s interesting to note that early research on the mouse complement system showed that, except for this strain, female mice have very low total complement activity (CH50); this is related to androgen regulation of hepatic complement synthesis and they don’t feel as anxious as men. Since C57BL/6 mice are the most popular inbred strain and provide remarkable genetic consistency, they are chosen above other murine models in *Schistosoma mansoni* research. In immunological and parasitological tests, repeatability and dependability are critical, and this consistency reduces biological variability among individual animals. They are especially useful for researching host-parasite interactions because of their well-characterized immune system and the experimentally manipulable Th1/Th2 polarization they display during schistosomiasis. Furthermore, C57BL/6 mice respond very well to vaccination procedures, such as gamma-irradiated cercariae vaccination, which generates robust T cell-mediated immunity and sheds light on defense mechanisms against schistosomiasis (Kotimaa et al. 2016 and El-Attar et al. 2024).

Praziquantel (PZQ) is the first-choice anthelminthic therapy due to its low cost and effectiveness against all schistosome species. It is unclear exactly how PZQ works. Following its administration, PZQ may interact with the hydrophobic ligand-binding pocket in the worm to activate the transient receptor potential ion channel. Voltage-gated calcium channels and pumps open as a result of this interaction, causing the worms to contract their muscles and become paralyzed as a result of the increased calcium ion inflow. Furthermore, PZQ exposes the parasite to the host’s immunological response by penetrating the worm’s tegument surface. Additionally, it prevents the worms from absorbing glucose, which immobilizes them and may even kill the parasites, although it does not completely cure drug resistance. Recently, *Schistosoma* worms have developed an immune defense against PZQ, so the total recovery became hard (Banda and Abere, 2025).

Numerous natural plants have demonstrated significant anti-schistosomal properties. The presence of bioactive substances such as terpenoids, alkaloids, flavonoids, and phenolic acids is linked to these effects. Furthermore, these compounds’ anti-schistosomal analysis revealed that they have significant activity against several targets. According to this review, natural plants are a valuable resource for the development of anti-schistosomal medications. To confirm their medicinal potential, more research on the toxicity of these bioactive chemicals and clinical studies are necessary (Balahbib et al. 2021).

*Ficus carica* (Fig) belongs to the *Moraceae* family. The GC mass of leaves contains ten essential compounds: phenolic compounds, alkaloids, saturated fatty acids, unsaturated fatty acids, and diterpene alcohol (El-Morsy et al. 2022). It has been used in traditional medicine to treat a variety of illnesses due to its numerous bioactivities, including antibacterial, antioxidant, anti-inflammatory, anticholinesterase, antidiabetic, anthelminthic, renoprotective, anticancer, and hepatoprotective properties (Mustafa et al. 2025). Extracts from Fig improved therapeutic performance, suggesting a synergistic effect that could help address medication resistance and partial parasite elimination (El-Attar et al. 2024). Simultaneously, medical reviews of *Ficus carica* emphasize its potent anti-inflammatory and antioxidant properties, specifically its ability to downregulate pro-inflammatory cytokines such as IL-1beta, IL-6, and TNF-alpha. Furthermore, comprehensive pharmacological profiles credit Fig extracts with high hepatoprotective and anticancer efficacy, driven by bioactive coumarins and flavonoids that induce cell cycle arrest and apoptosis (Fazel et al. 2024). From an oncology perspective, medical literature confirms that Fig-derived compounds-particularly those found in the latex and leaves- suppress cancer cell proliferation and induce mitochondrial-mediated apoptosis in breast, liver, and colorectal carcinoma models (Gökçen et al. 2026; Fazel et al. 2024).

*Olea europaea* L. (Olive) belongs to the family *Oleaceae*. Its fruit has been found to contain more than 30 phenolics. Oleuropein β-(3,4-dihydroxyphenylethanol) or hydroxytyrosol and p-hydroxyphenylethanol (tyrosol) are the primary phenolic alcohols. Additionally, Flavonol glycosides, including luteolin 7-O-glucoside, rutin, apigenin 7-O-glucoside, anthocyanins, cyanidin 3-O-glucoside, and cyanidin 3-O-rutinoside, make up the majority of the flavonoid chemicals. Gallic acid, protocatechuic acid, p-hydroxybenzoic acid, vanillic acid, caffeic acid, coumaric, cinnamic, and ferulic acid are the main polyphenols (Hussain et al. 2021). β-carotene, lutein, and zeaxanthin are the carotenoids found (Ayoub et al. 2019). Olive leaf extracts are clinically recognized for their cardiometabolic benefits, with reviews of randomized clinical trials showing significant improvements in blood pressure, lipid profiles, and glucose tolerance. Mechanistically, Olive compounds like oleuropein protect against neurodegeneration by inhibiting beta-amyloid aggregation and reducing neuroinflammation via the NF- kappa B pathway (Ben-Amor et al. 2021; Gökçen et al. 2026). These active compounds are effective as used in antioxidant, anti-inflammatory, anti-ageing, anti-cancer, anti-schistosomal, neuroprotector, and anti-diabetic (Talib et al. 2024).

Extracts from Olive have demonstrated significant antischistosomal properties against *Schistosoma mansoni*. In vitro, extracts from Olive leaves, which are high in phenolic compounds like oleuropein, have shown strong schistosomicidal action, killing adult worms in a dose-dependent manner. Olive extracts improved intestinal and hepatic pathologies, decreased worm load, and decreased egg deposition in infected mice. Direct antiparasitic benefits, antioxidant activity that reduces oxidative stress brought on by infection, and immunomodulatory properties that improve host defense responses are some of the suggested methods. According to these results, PZQ may benefit from the use of olive extracts as a supplemental therapy, which could enhance treatment results and lower the potential of drug resistance (Reda et al. 2016).

The manipulation of atoms and molecules at the nanoscale is referred to as nanotechnology. It can change a substance’s chemical and physical characteristics by altering it at the atomic level. The nano-capsules can deliver existing drugs to their target. Nano-capsules should allow as much as a 10.000-fold decrease in drug dosages, reducing the harmful side effects of drugs used in chemotherapy. There are numerous uses for green nanoparticle production in the biomedical and environmental domains. One of the main goals of green synthesis is to use fewer harmful chemicals. For example, using organic materials, like plants, is generally safe. Additionally, plants contain capping and reducing agents. Gold, silver, copper, palladium, platinum, zinc oxide, and titanium dioxide are examples of nanoparticles. The use of silver nitrate in the preparation of nano capsules of Fig was evaluated (*Ficus carica*-silver loaded nanoparticles) and used in the treatment of infected mice with *S. mansoni*, which did not show toxicity, while it recorded a cure effect rather than Fig nanoparticles alone (El-Attar et al. 2024).

The presented study was evaluated to assess the effect of Fig and Olive nano capsules on C57BL/6 mice infected with *Schistosoma mansoni* at all different stages of the life cycle.

## Materials

### Drugs

Praziquantel (PZQ) tablets were purchased from the Egyptian International Pharmaceutical Industries Company, Mansoura, Egypt.

### Plants

In May 2025, leaves of Fig and Olive were collected locally from their natural habitats in Egypt, especially in the Delta region. Both plants were legitimized by the Taxonomy Professors of the Botany Department, Faculty of Science, Mansoura University, Mansoura, Egypt.

All other chemicals used in this thesis were highly purified to an analytical grade.

### Malvern Zetasizer nano instrument

Malvern Panalytical developed the Malvern Zetasizer Nano, a scientific device for determining the molecular weight, size, and zeta potential of particles, molecules, and colloids in liquid suspension. It is extensively utilized in both industry and research, particularly in the fields of biochemistry, materials science, nanotechnology, and medicine. Sample Requirements: Small volumes (typically less than 1 mL), aqueous or non-aqueous dispersions, and minimal sample preparation for most measurements (Bhattacharjee 2016).

This instrument was used to measure the size and potential of Fig and Olive nanoparticles at 25 °C in the Electron Microscope Unit, Faculty of Agriculture, Mansoura University, Mansoura, Egypt.

### Experimental animals and parasites

C57BL/6 black female mice aged 6–7 weeks with an average weight of 18–19 g were obtained from the Merc Institute, Faculty of Medicine, Mansoura University, Mansoura, Egypt. Mice were acclimated in wood-chip bedding plastic cages, refreshed every day for a week. They were supplied with standard food, consisting of pellets of commercial rodent food, and water *ad libitum* during the experimental period, at 22 ± 3 °C, with a 12-h cycle of darkness and 12 h of light in the animal house of the Faculty of Science, Mansoura University, Mansoura, Egypt.

An Egyptian strain of *Schistosoma mansoni* cercariae was obtained from freshly shedding infected intermediate host snails, *Biomphalaria* a*lexandrina,* which were maintained at the Theodor Bilharz Research Institute, Cairo, Egypt.

### Experimental groups and mode of administration

Forty-two C57BL/6 female mice were kept for the experimental design of seven groups (6 mice/group) as follows:

1. *Negative control group (–ve control)* Uninfected and untreated.
2. *Positive control group (+ ve control)* Infected- untreated.
3. *Praziquantel group (PZQ)* An oral dosage of 200 mg/kg was administered for two consecutive days at the beginning of the 6th week following infection (El-Lakkany et al. 2011).
4. *Administered by the Fig and Olive nanoparticles group at the Cercariae stage (G4)* 400 mg/kg, orally taken on the day of infection; mice were administered with Fig nano-capsules after 4 h of infection, then after another 2 h, Olive nanoparticles (400 mg/kg) were taken.
5. *Administered by the Fig and Olive nanoparticles group at the Schistosomulae stage (G5)* 400 mg/kg, orally taken on the 4th day of infection; mice were given Fig nanoparticles, then, after another 2 h, Olive nanoparticles (400 mg/kg) were taken.
6. *Administered by the Fig and Olive nanoparticles group at the Immature worm stage (G6)* 400 mg/kg, administered orally on the 11th day of infection; mice received Fig nanoparticles, followed by the intake of Olive Olive nanoparticles after an additional 2 h (400 mg/kg).
7. *Administered by the Fig and Olive nanoparticles group at the Mature worm stage (G7)* 400 mg/kg was administered orally on the first day of the sixth week post-infection; mice received Fig nanoparticles, followed by 400 mg/kg of Olive nanoparticles after an additional 2 h.

The concentration and dosage of Fig extract nanoparticles utilized in the studies by El-Morsy et al. (2022) and El-Attar et al. (2024). The given concentration of Olive extract nanoparticles was estimated by Reda et al. (2016). Both were based on previous experimental studies made by El-Shabasy, E. A..

The dosage time was picked up based on the *Schistosoma mansoni* life cycle recorded by Parija and Chaudhury (2022).

## Methods

### Ethical proof

The experimental protocol was conducted in accordance with the guidelines of the National Institutes of Health for the care and use of laboratory animals (NIH publication No. 8523, revised 1996) and was approved by the Mansoura University-Animal Care and Use Committee (MU-ACUC), with approval number Sc. PhD. 25.6.35. The date of approval is 1/6/2025.

#### Infection of mice

All mice groups, excluding the negative control group, were injected subcutaneously with freshly shed cercariae from a stock solution that primarily consisted of 70 cercariae in 0.5 ml of distilled water.

#### Preparation of the praziquantel fresh solution

Two hundred milligrams of PZQ were combined with a single drop of highly purified olive oil until a paste-like consistency was achieved, after which 1 ml of sterile water was incrementally added while continuously stirring.

#### Preparation of powdered leaves of both plants

Fig and Olive leaves were meticulously washed until they were entirely free of impurities; the drying process took place for 2 weeks in a shaded environment at room temperature, with the leaves being turned continuously; subsequently, they were ground into a fine powder using an electric grinder from Sokany Company, located in Cairo, Egypt.

#### Preparation of plants extracts nanoparticles

Ten milliliters of ethanolic extracts from Fig and Olive were combined with 10 ml of AgNO_3_, continuously stirred for one hour at a pH of 7, then allowed to sit in the dark for 24 h. After this period, the mixture was stirred once more and left until a yellowish-green color was observed in the Fig extract or a dark brown color was noted in the Olive extract.

#### Characterization of nanoparticles

The Malvern Zetasizer employed the dynamic laser light scattering method to evaluate the hydrodynamic zeta potential and particle size of nanoparticles for Fig and Olive ethanolic extracts (Ulaeto et al. 2020).

After the experiment, mice were administered subcutaneously with cercariae (70–75) following the procedure outlined by Liang et al. (1987). The mice were monitored for 5 weeks (Peters and Warren, 1969) before receiving treatment with PZQ for 2 consecutive days during the 6th week, after which all mice underwent scarification at the end of the 7th week.

#### Blood sampling

After a duration of 7 weeks of infection, the mice were anesthetized using ketamine (43.5 mg/kg) and xylazine (6.5 mg/kg) for a period of 35 mins (El-Sherif 2019). Following this, the mice were sacrificed, and blood samples were obtained in sterile centrifuge glass tubes. These samples were allowed to clot completely before being centrifuged at 1350×*g* for 15 mins. The clear supernatant was then swiftly collected. The serum was stored in appropriately labeled Eppendorf tubes and frozen at −20 °C for subsequent biochemical analyses.

#### Liver tissue preparation

Livers from mice were collected, and the same lobe was obtained from each mouse, which was then frozen at − 20 °C for various assessments.

#### Hepatic tissue homogenate preparation

Liver samples weighing 1 g were homogenized to a concentration of 10% w/v using distilled water at a force of 1350×*g* for a duration of 20 mins at a temperature of 4 °C; the resulting homogenates were subsequently stored in appropriately labeled Eppendorf tubes at −20 °C for biochemical analyses, immunological assessments, and comet assay.

#### Immunological assessments

##### Cytokine profile

Hepatic interleukin-4 (IL-4) (pg/mg), interleukin-5 (IL-5) (pg/mg), interleukin-10 (IL-10) (pg/mg), interleukin-13 (IL-13) (pg/mg) proteins were evaluated using a Rat IL-4 ELISA Kit, Rat IL-5 ELISA Kit, Rat IL-10 ELISA Kit, Rat IL-13 ELISA Kit, respectively from ELISA Kit, San Diego, California, USA, also, interleukin-6 (IL-6) (pg/mg) and Transforming Growth Factor β (TGF-β) (pg/mg) from ELISA Kit and Rat Transforming Growth Factor β (TGF-β) ELISA Kit from CUSABIO, Houston, TX, USA, with catalog numbers MBS2503139, MBS2503349, MBS355232, MBS355408, CSB-E04640r, and CSB-E04727r, respectively. The genome’s coding is PRJEA36577, 24497, PRJEA36577, PRJEA36577, 21/308, and 24899, respectively.

##### Antibody production

The hepatic immunoglobulin E (IgE) (ng/mg) concentration was estimated by using Rat Immunoglobulin E (IgE) ELISA Kit from CUSABIO, Houston, TX, USA, in addition to immunoglobulin G1 (IgG1) (ng/mg), and immunoglobulin G2 (IgG2) (ng/mg) concentrations were evaluated by the aid of Rat Immunoglobulin G1 (IgG1) ELISA Kit and Rat Immunoglobulin G2 (IgG2) ELISA Kit from MyoBioSource, San Diego, California, USA, with catalog numbers CSB-E07984r, MBS2020351, MBS2515988, respectively were used to measure. The coding numbers of the genome are 289827, PRJEA36577, and PRJEA36577, respectively.

#### Biochemical markers

##### Oxidative stress markers and antioxidants

Hepatic malondialdehyde (MDA) (nmol/g), nitric oxide (NO) (µmol/g), glutathione (GSH) (mmol/g) contents, superoxide dismutase (SOD) (U/g), and catalase (CAT) (U/g) activities were evaluated according to the method described by Ohkawa et al. (1979); Montgomery and Dymock (1961); Beutler et al. (1963); Nishikimi et al. (1972); Aebi (1984), respectively. The use of a colorimetric kit from Biodiagnostic and research reagents, Dokki, Giza, Egypt, with a catalog number MD 25 29, NO 25 33, GR 25 11, SD 25 21, CA 25 17, respectively. The coding number of the genome is 100683-54-3, D00074, 104090, R01 CA073599, and 1.11.1.6, respectively.

##### Inflammatory markers

Serum C-reactive protein (CRP) in ng/ml content was evaluated according to the method described by Banerjee et al. (2003), using Rat C-Reactive Protein [CRP] ELISA Kit, San Diego, California, USA. Hepatic vascular cell adhesion molecule-1 (VCAM-1) (ng/mg), and intercellular adhesion molecule-1 (ICAM-1) (pg/mg) contents were measured using an ELISA kit and Rat Intercellular Adhesion Molecule 1 (ICAM-1) ELISA Kit from CUSABIO, Houston, TX, USA. The catalog numbers are 557825, CSB-E07275r, and CSB-E04576r, respectively. The codes of genomes are PRJEA36577, 7412, and 25464, respectively.

##### Apoptotic and anti-apoptotic markers

Hepatic tumor protein 53 (P53) (pg/mg) and cytochrome-C (pg/mg) concentrations were measured using a Rat P53/Tumor Protein (P53/TP53) ELISA Kit and an ELISA kit, CUSABIO Innovation Center, Houston, TX, USA. Bcl-2-like protein 4 (Bax) in pg/mg and Cysteine-aspartic proteases-8 (Caspase-8) (ng/mg) contents were estimated by using Rat apoptosis regulator BAX (BAX) ELISA Kit and Rat Caspase-8 (Caspase-8) ELISA Kit, MyBioSource, San Diego, California, USA. Rat B-cell CLL/lymphoma 2 (BCL2) ELISA Kit from Creative Bio Laboratories, Ramsey Road, Shirley, USA was used to estimate hepatic B-cell lymphoma 2 (Bcl-2) content (ng/mg). Hepatic Cysteine-aspartic proteases-9 (Caspase-9) (pg/mg) and Cysteine-aspartic proteases-3 (Caspase-3) (ng/mg) contents were estimated by an ELISA kit from Rat Caspase-9 (Casp-9) ELISA Kit and ELISA kit, CUSABIO Innovation Center, Wuhan, Hubei Province, China. The catalog numbers are CSB-E08336r, CSB-EL006328RA, MBS935667_48T, MBS260539, MBS704498, CSB-E08863r, and CSB-E08857r, respectively. The genomes’ codes are ENST00000269305.9_8, 54205, 25212, PRJEA36577, 0000278, 29153, and PRJEA36577, respectively.

##### Liver function tests

Serum Alanine aminotransferase (ALT) (U/L), Aspartate aminotransferase (AST) (U/L), Alkaline phosphatase (ALP) (U/L), Gamma-glutamyl transferase (ɤ-GT) (U/L) activities, Albumin (g/dl) and Bilirubin (mg/dl) contents have been measured using the Spinreact kit, La Vall d’en Bas, Girona, Spain, with catalog numbers 1001172, MD41264, 41246, MD41288, MX1001020, MD1001042, respectively, in accordance with the Young (2001) colorimetric kit method. The genome has the code 41210, 41220, 41242, 41230, 001081, 001099, respectively.

##### The Comet assay

The crushed samples (0.5 g) were placed into 1 ml of ice-cold phosphate-buffered saline (PBS). This suspension was stirred for 5 min and subsequently filtered. A cell suspension of 100 μl was combined with 600 μl of low-melting agarose (0.8% in PBS). A volume of 100 μl from this mixture was applied to pre-coated slides. The coated slides were then submerged in a lysis buffer (0.045 M Tris/Borate/EDTA (TBE), pH 8.4, containing 2.5% Sodium dodecyl sulfate (SDS)) for 15 min. Finally, the slides were transferred into an electrophoresis chamber filled with the same TBE buffer, but without SDS.

The conditions for electrophoresis were set at 2 V/cm for 2 min and a current of 100 mA. Staining was performed using ethidium bromide at a concentration of 20 μg/ml at a temperature of 4 °C. Observations were conducted while the samples remained moist, and the DNA fragment migration patterns of 100 cells for each dose level were assessed using a fluorescence microscope (with an excitation filter of 420–490 nm and an emission filter of 510 nm). The lengths of the comet tails were measured from the center of the nucleus to the end of the tail using a 40 × magnification to count and determine the size of the comet. For the visualization of DNA damage, observations were made of the EtBr-stained DNA utilizing a 40 × objective on a fluorescent microscope.

While any image analysis system could potentially be appropriate for quantifying single-cell gel electrophoresis assay data, the Komet 5 image analysis software, created by Kinetic Imaging, Ltd. (Liverpool, UK), is connected to a CCD camera. This setup allows us to evaluate both the quantitative and qualitative levels of DNA damage in the cells by measuring the length of DNA migration and the percentage of DNA that has migrated. Ultimately, the software computes the tail moment (Singh et al. 1988). Typically, between 50 and 100 randomly chosen cells are analyzed for each sample (Tice et al. 2000).

### Statistical analyses

All statistical data were prepared using GraphPad Prism 8.0 (GraphPad Software Inc., San Diego, California, USA). The mean ± standard deviation (SD) (n = 6) is displayed for the results. According to Armitage et al. (2008), statistical contrasts were created using a one-way analysis of variance (ANOVA), followed by the Neuman-Keuls Post-hoc test. *P* < 0.05 is the significance level.

## Results

### Characterization of nano-samples

#### Zeta potential and size of Fig nanoparticles

**Fig. 2:**
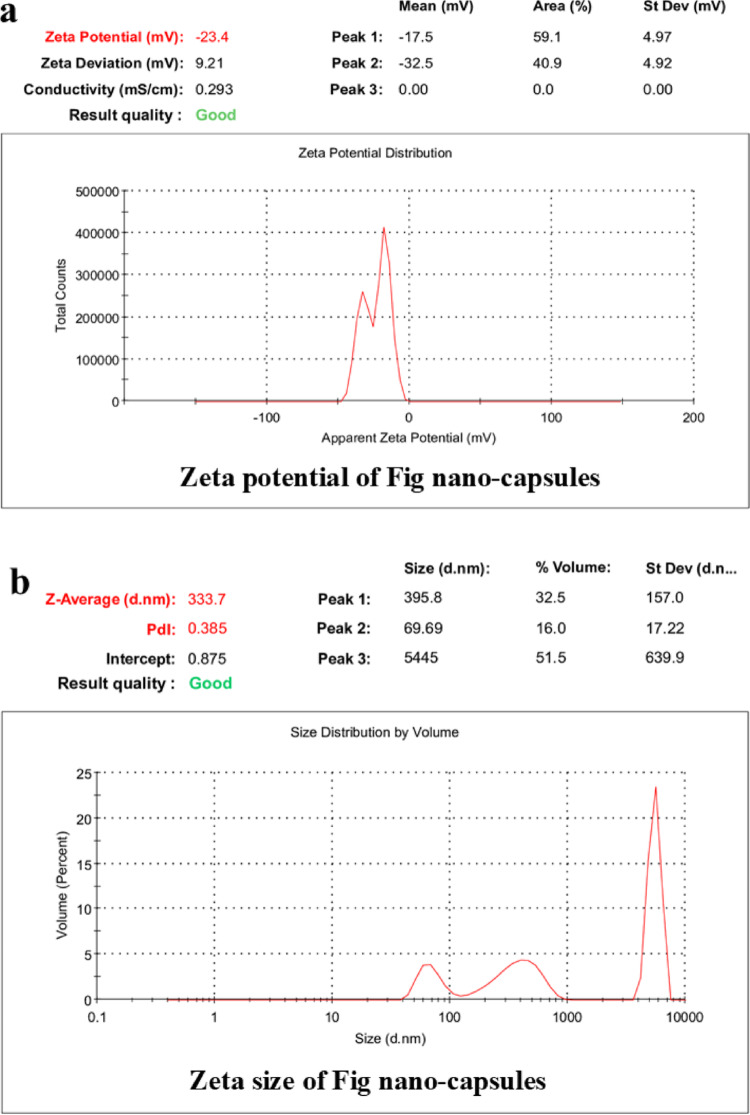
Characterization of Fig nanoparticles as **a** Zeta potential and **b** Size of nanoparticles

The charge of Fig nanoparticles recorded a mean voltage of −23.4 mV as observed in Fig. 2a. Their average diameter was recorded as 333.7 nm, as recorded in Fig. 2b.

#### Zeta potential and size of Olive nanoparticles

**Fig. 3:**
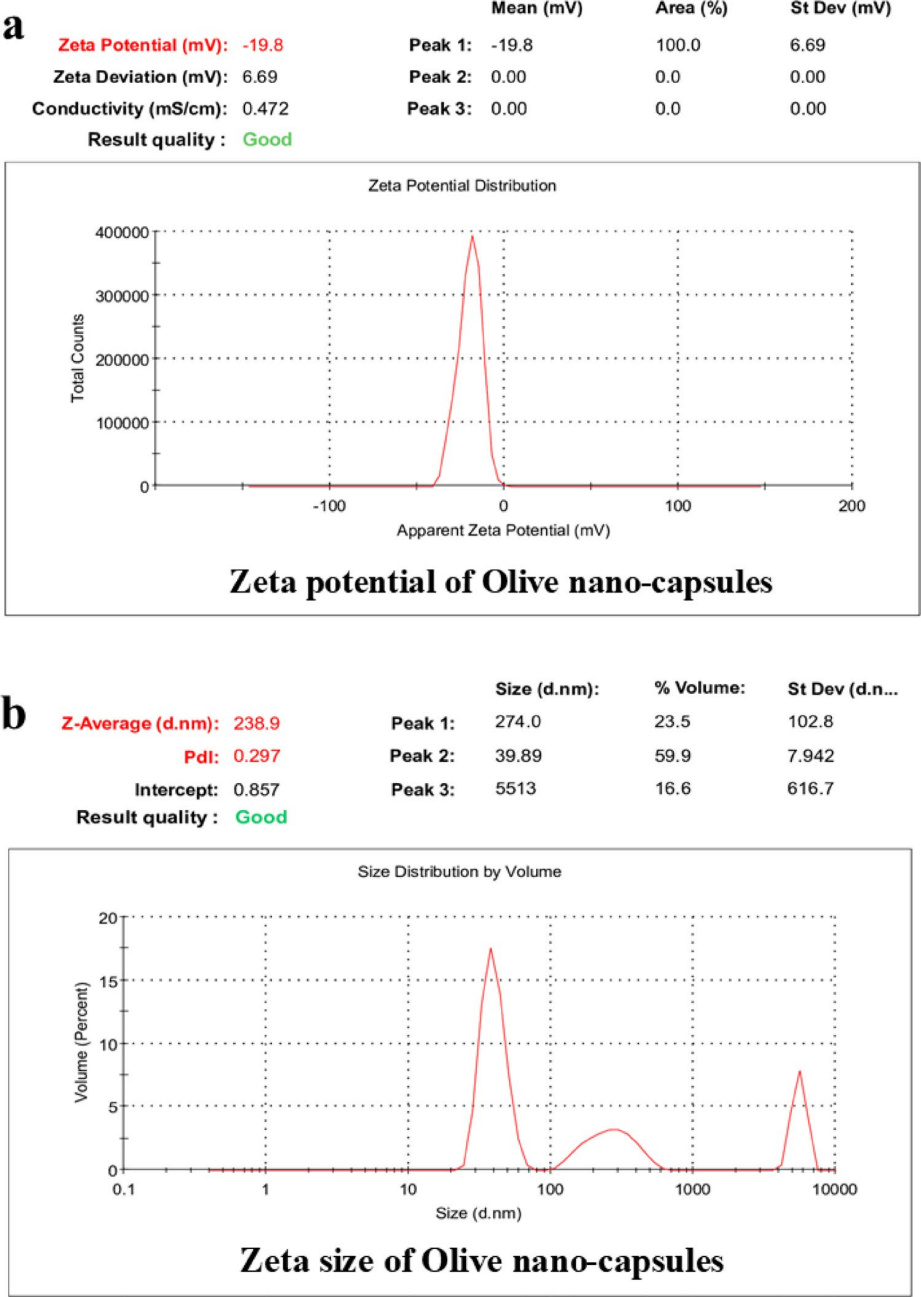
Characterization of Olive nanoparticles as **a** Zeta potential and **b** Size of nanoparticles

The charge of Olive nanoparticles had a mean voltage of −19.8 mV as observed in Fig. 3a. Their average diameter was recorded at 238.9 nm, as shown in Fig. 3b.

### Immunological assessment

#### Cytokine profile

**Fig. 4:**
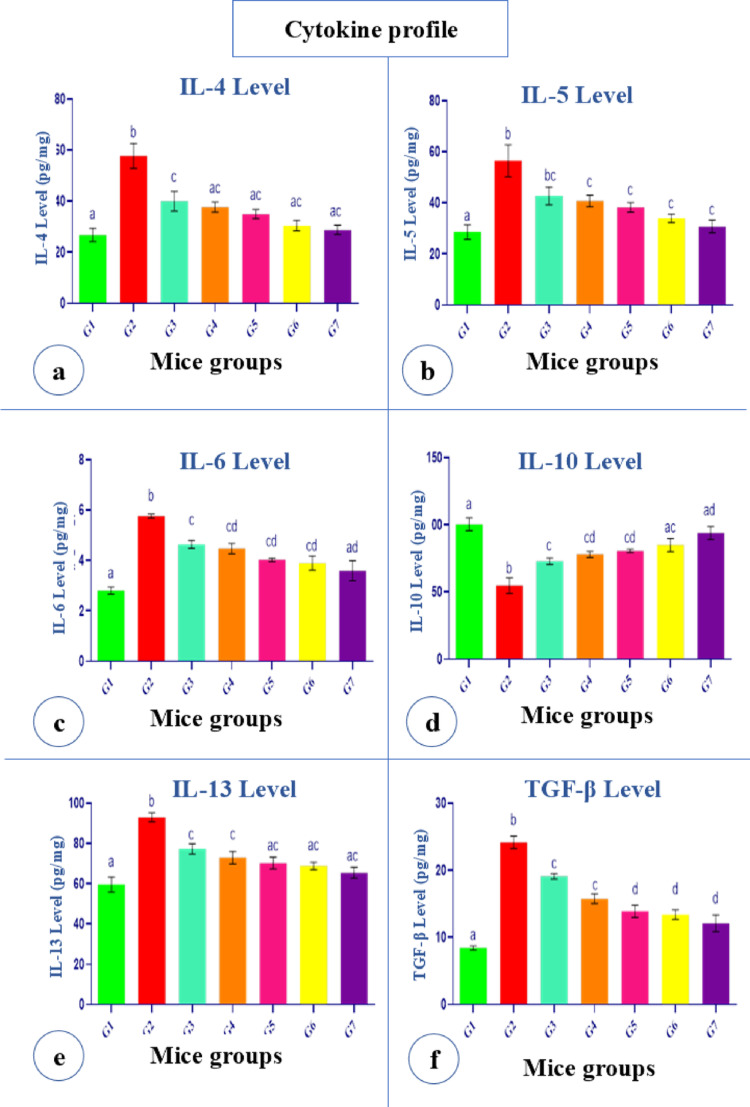
Cytokine profile; **a** Interleukin-4, **b** Interleukin-5, **c** Interleukin-6, **d** Interleukin-10, **e** Interleukin-13, and **f** Transforming growth factor β

Figure 4a–f observed cytokine profile represented in interleukin-4 (IL-4), interleukin-5 (IL-5), interleukin-6 (IL-6), interleukin-10 (IL-10), interleukin-13 (IL-13), and transforming growth factor-β (TGF-β) levels, respectively, which were evaluated in the hepatic homogenate of control and different treated groups.

The data obtained showed that IL-4, IL-6, IL-13, and TGF-β levels were significantly decreased in G3, although IL-5 level was not. Additionally, a significant increase in IL-10 level was noted. The G4, G5, G6, and G7, respectively, recorded a significant decrease in IL-4, IL-5, IL-6, IL-13, and TGF-β levels; in contrast, a significant increase in IL-10 level compared with G2. Among all groups, G7 exhibited the most effective modulation of the cytokine profile, indicating superior therapeutic efficacy at the mature worm stage.

#### Antibody production

Figure 5a–c observed antibody production represented in immunoglobulin E (IgE), immunoglobulin G1 (IgG1), and immunoglobulin G2 (IgG2) levels, which were estimated in the hepatic tissue of control and various treatment groups.

**Fig. 45:**
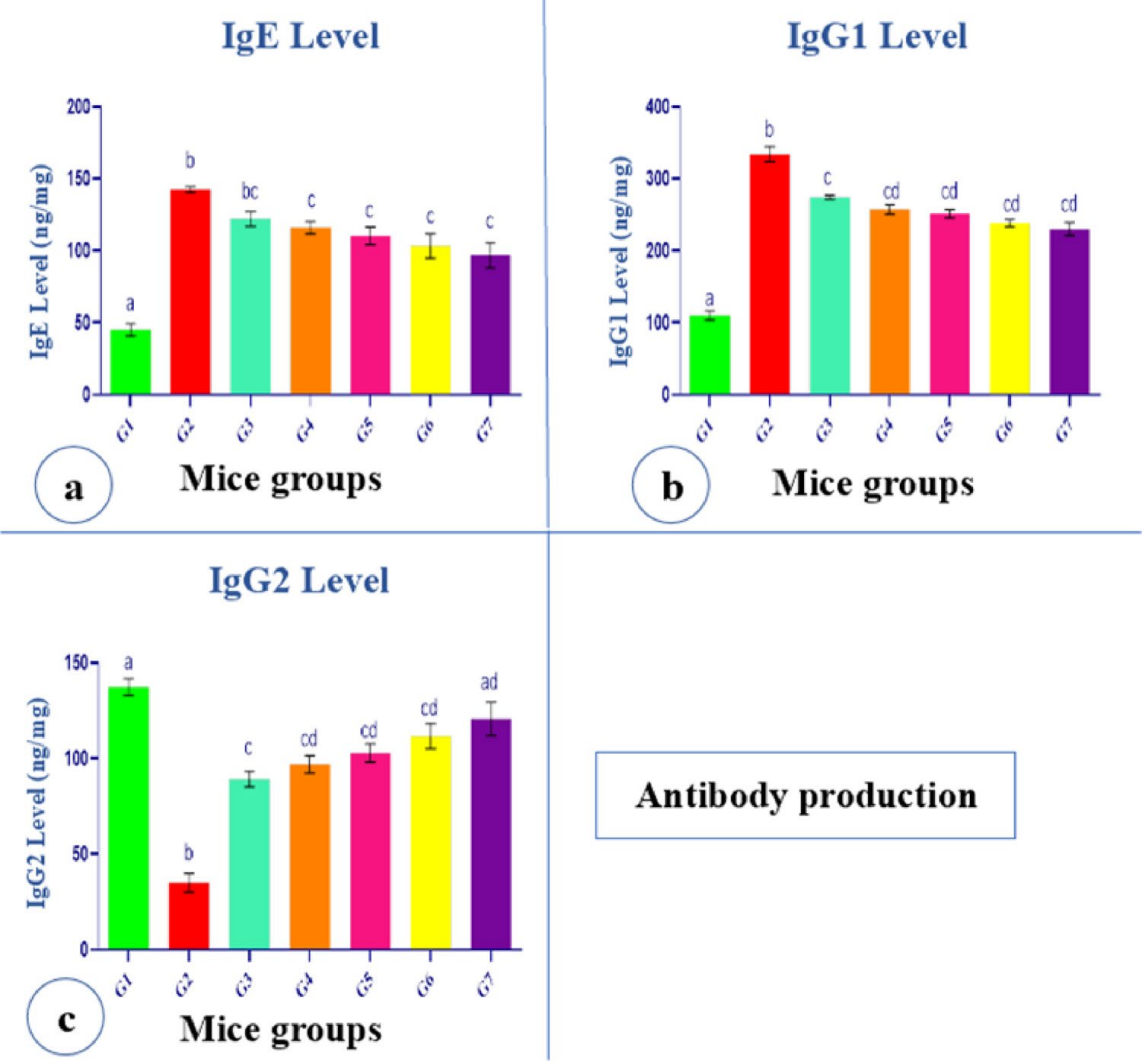
Antibody production; **a** Immunoglobulin E, **b** Immunoglobulin G1, **c** Immunoglobulin G2

The IgE and IgG1 levels were significantly decreased in G3, although the IgG2 level was significantly increased. The G4, G5, G6, and G7, respectively, showed a significant decrease in IgE and IgG1 levels, in addition to a significant increase in IgG2 level, compared with G2. The G7 showed the best group in regaining the antibody production.

### Biochemical markers

#### Oxidative stress markers and antioxidants

**Fig. 6:**
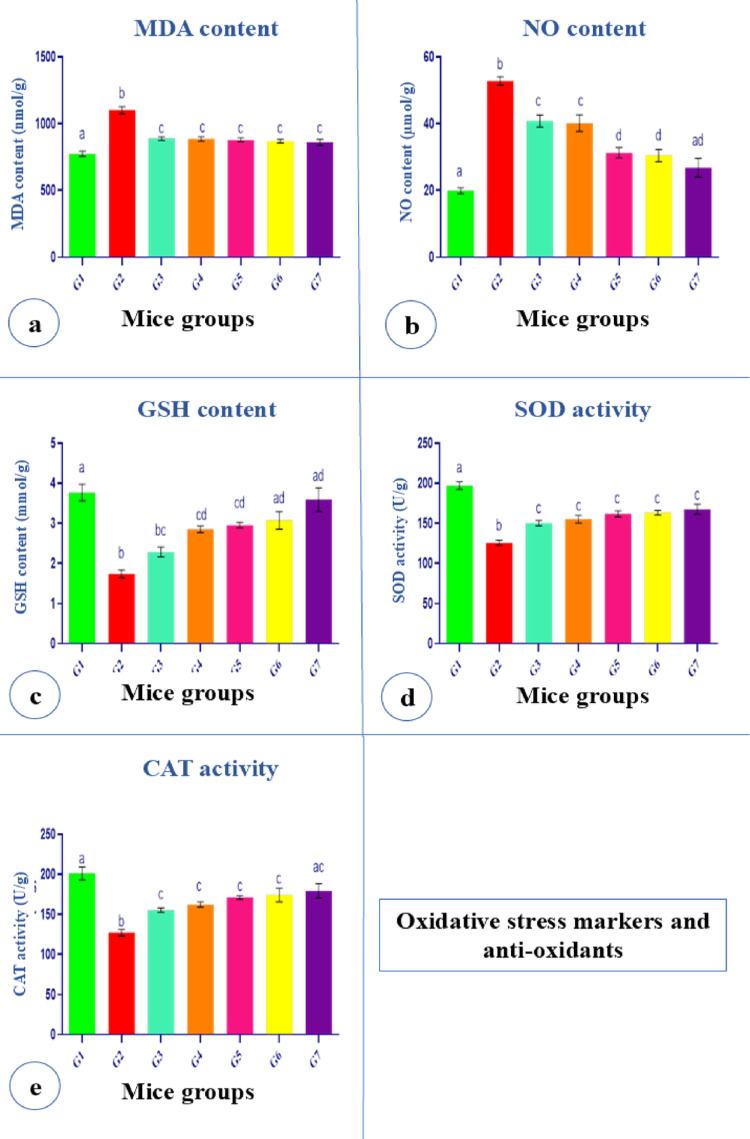
Oxidative stress markers and antioxidants; **a** Malondialdehyde, **b** Nitric oxide, **c** Glutathione, **d** Superoxide dismutase, and **e** Catalase

Figure 6a–e observed oxidative stress markers and antioxidants as Malondialdehyde (MDA), Nitric Oxide (NO), Glutathione (GSH) contents, Superoxide dismutase (SOD), and Catalase (CAT) activities, which were evaluated in the hepatic homogenate of control and different treated groups.

The G3 showed a significant decrease in MDA and NO contents, in addition to an observed significant increase in SOD and CAT activities, unlike an insignificant increase in GSH activity. The G4, G5, G6, and G7, respectively, showed a significant decrease in MDA and NO contents and a significant increase in GSH level, SOD, and CAT activities compared with G2. The G7 showed the most effective group in the improvement of oxidative stress and antioxidants.

#### Inflammatory markers

**Fig. 7:**
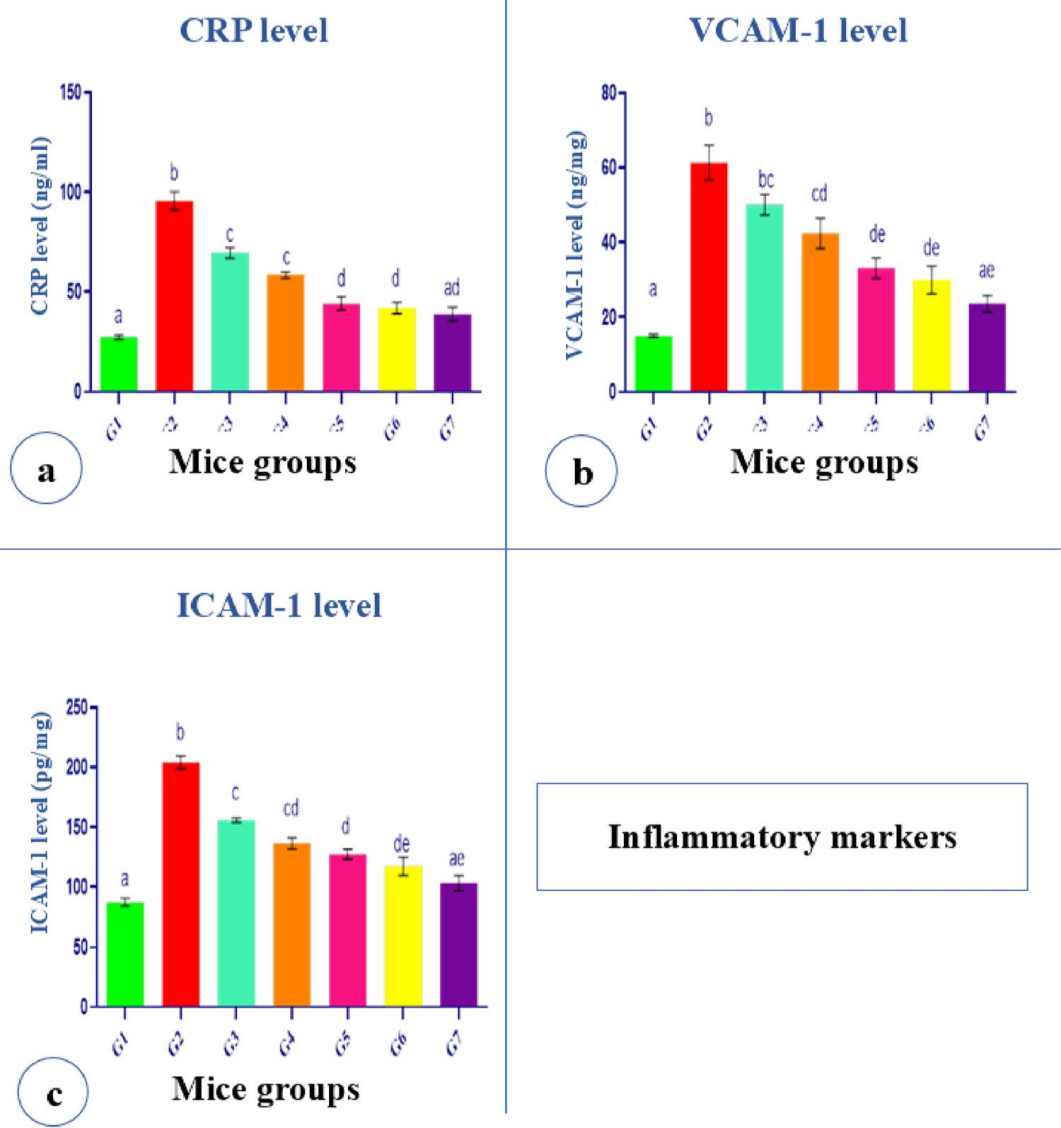
Inflammatory markers; **a** C-reactive protein, **b** Vascular cell adhesive molecule-1, **c** Intercellular adhesive molecule-1

Figure 7a–c observed inflammatory markers as C-reactive protein (CRP), Vascular cell adhesive molecule-1 (VCAM-1), Intercellular adhesive molecule-1 (ICAM-1) levels, which were evaluated in the hepatic homogenate of control and different treated groups.

The G3 recorded a significant decrease in CRP, VCAM-1, and ICAM-1 compared with G2. The G7 showed the best group in the decrease of inflammatory markers.

#### Apoptotic and anti-apoptotic markers

**Fig. 8:**
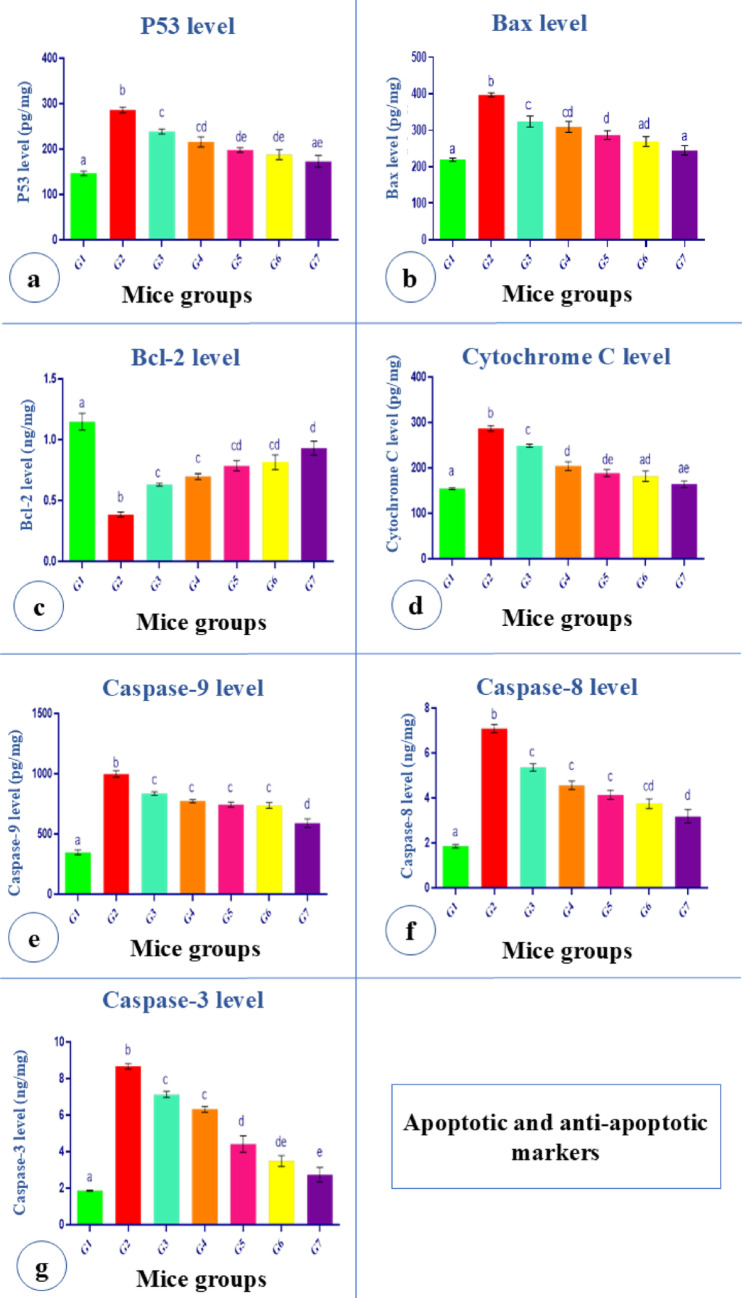
Apoptotic markers and anti-apoptotic; **a** tumor protein 53, **b** bcl-2-like protein 4, **c** B-cell lymphoma 2, **d** Cytochrome C, **e** Cysteine-aspartic proteases-9, **f** Cysteine-aspartic proteases- 8, **g** Cysteine-aspartic proteases-3

Figure 8a–g observed apoptotic and antiapoptotic markers as tumor suppress protein tumor protein 53 (P53), bcl-2-like protein 4 (Bax), B-cell lymphoma 2 (Bcl-2), Cytochrome C, Cysteine-aspartic proteases 9 (Caspase-9), Cysteine-aspartic proteases 8 (Caspase-8), Cysteine-aspartic proteases 3 (Caspase-3) levels, which were evaluated in the hepatic tissue of control and different treated groups.

The G3 recorded a significant reduction in P53, Bax, Cytochrome C, Caspase-9, Caspase-8, and Caspase-3 contents in comparison to G2, but a significant elevation in Bcl-2 content. The G4, G5, G6, and G7, respectively, recorded significant decrease in P53, Bax, Cytochrome C, Caspase-9, Caspase-8, and Caspase-3 contents; unlike, significant increase in Bcl-2 content compared with the G2 group. The G7 was the highest suppressant in the apoptosis signaling process.

#### Liver function tests

**Fig. 9:**
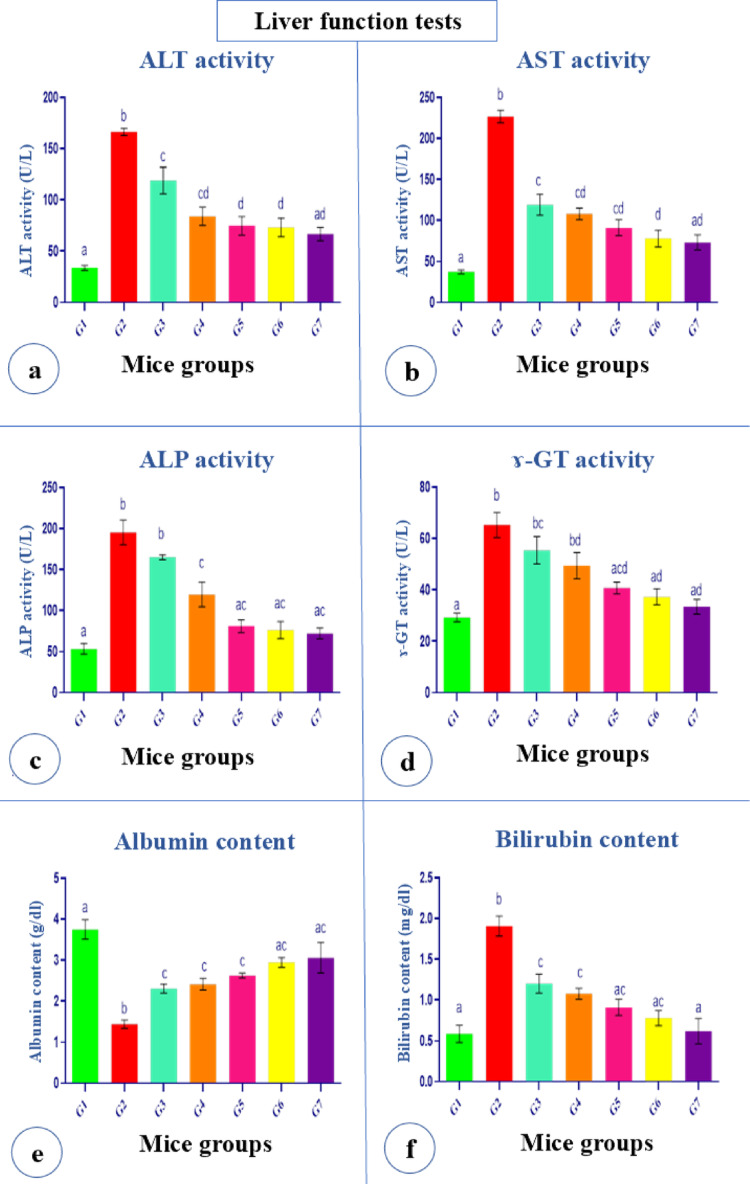
Liver function tests; **a** Alanine aminotransferase, **b** Aspartate aminotransferase, **c** Alkaline phosphatase, **d** Gamma-glutamyl transferase, **e** Albumin, and **f** Bilirubin

Figure 9a–f showed liver function markers represented in alanine aminotransferase (ALT), aspartate aminotransferase (AST), alkaline phosphatase (ALP), gamma-glutamyl aminotransferase (ɤ-GT) activities, albumin, and bilirubin contents, which were estimated in the serum of control and various treated groups.

The G3 showed a significant decrease in ALT, AST activities, and bilirubin content, while a non-significant decrease in ALP and ɤ-GT activities. It observed a significant increase in albumin content. The G4, G5, G6, and G7, respectively, showed significant decreases in ALT, AST, ALP, and ɤ-GT activities and in bilirubin content, although a significant increase in albumin compared with G2.

#### DNA damage assessments

Figure 10a–c illustrated tail length, Tail moment, and Tail DNA levels, respectively, which were assessed in the hepatocytes of control and various treated groups, as observed in Fig. 11a–g. The G3, G4, G5, G6, and G7, respectively, showed significant reduction in Tail length, Tail moment, and Tail DNA contents, observing a reduction in DNA damage tailed spots in Fig. 11 compared with G2.

**Fig. 10:**
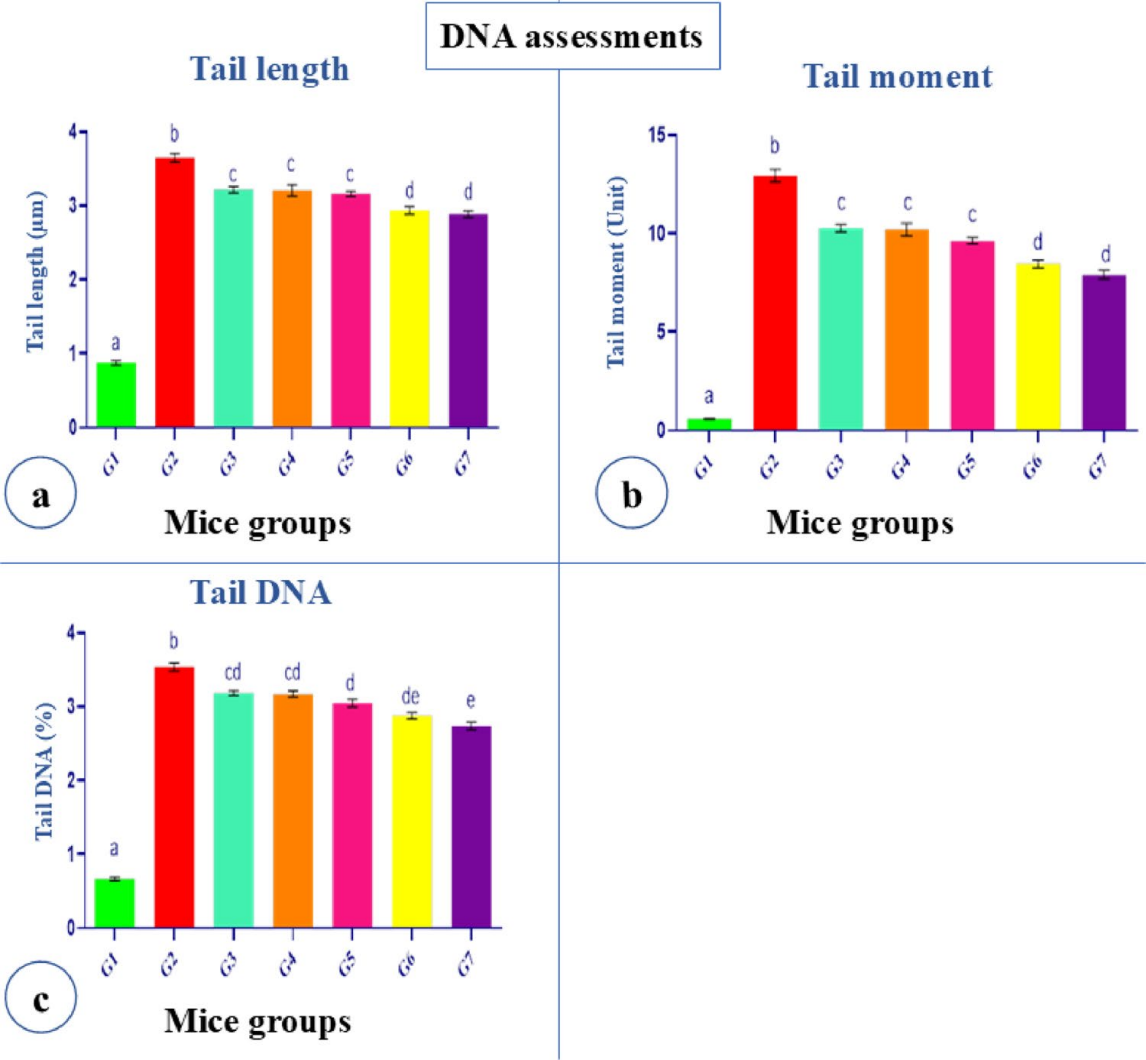
DNA damage assessments in the hepatocytes of control and different treated mice groups; **a** Tail length, **b** Tail moment, and **c** Tail DNA

**Fig. 10:**
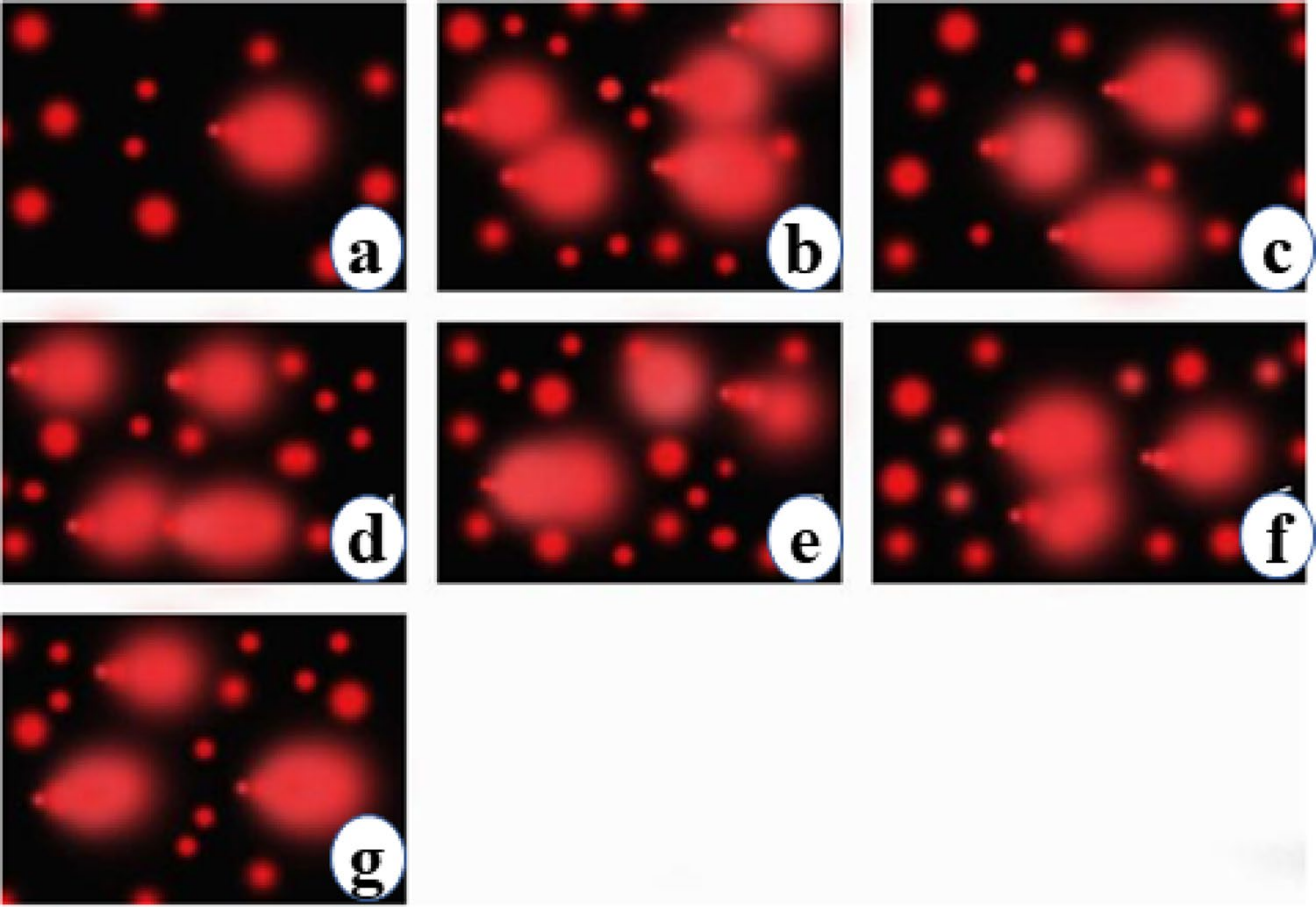
Representative photomicrographs of the comet assay on hepatic cells

## Discussion

Both plants were freshly prepared and were just measured by zeta-potential and zeta-size. The nanoparticle morphology (e.g., TEM or SEM images), particle size distribution, nanoparticle stability, and encapsulation efficiency or loading capacity are estimated and will be published in the next paper.

Infection with *Schistosoma mansoni* in murine models triggers a complex pathological sequence characterized by profound biochemical and immunological shifts centered primarily in the liver. As parasite eggs become trapped in hepatic tissue, their toxins induce a significant increase in oxidative stress, marked by elevated MDA levels and a concomitant depletion of antioxidant markers such as GSH and SOD. This oxidative environment exacerbates the inflammatory response, driving increases in inflammatory markers (e.g., TNF-alpha and CRP) and triggering apoptotic markers (e.g., Caspase-3), which contribute to hepatocyte death, as evidenced by deranged liver function tests, including elevated ALT and AST (Gryseels et al. 2006).

The immunological assessment of this infection reveals a dominant Th2-polarized response; the host displays significantly elevated levels of IL-4, IL-5, IL-6, IL-10, and IL-13, which collectively drive granuloma formation and tissue remodeling. This cytokine milieu is often accompanied by a rise in TGF-β, a key mediator of hepatic fibrosis. Systemically, this shift is mirrored in the humoral response, with marked elevations in IgE, IgG1, and IgG2 levels, reflecting the chronic attempt of the immune system to manage the persistent helminthic burden (El-Lakkany et al. 2012).

In a murine model, PZQ in the G3 group observed significantly lower amounts of IL-4, IL-6, IL-13, and TGF-β, but not significantly lower levels of IL-5. There was a noticeable increase in IL-10 content. The IgE level was not significantly reduced, while the IgG1 level was. There was a significant rise in IgG2 content also revealed a significant increase in SOD and CAT activities with a significant reduction in MDA and NO contents, in contrast to an insignificant rise in GSH activity. CRP and ICAM-1 levels were found to have significantly decreased; however, VCAM-1 level was found to have decreased insignificantly. It showed a significant change in apoptotic markers; a rise in Bcl-2 content but a significant decrease in P53, Bax, Cytochrome C, Caspase-9, Caspase-8, and Caspase-3 levels. It observed a significant reduction in Tail length, Tail moment, and Tail DNA%. Its ALT, AST activities, and bilirubin content were significantly lower, although ALP and ɤ-GT activities were not, while a notable increase in albumin content was observed compared with G2.

All the previous observations were matched with Abdel-Hafeez et al. (2012); Kong et al. (2018); Nono et al. (2021); Fassi et al. (2022); de Lima Aires et al. (2023) and Abebe et al. (2025). They explained that PZQ successfully eliminates adult worms and reduces egg burden when used to treat *S. mansoni* infection. This suppresses antigen-driven Th2 cytokines (such as IL-5 and IL-13), but paradoxically, it can also be linked to persistent or even elevated TGF-β, which causes fibrosis. By raising GSH and lowering MDA, PZQ partially restores redox balance; nevertheless, antioxidant enzymes like SOD and CAT might not fully recover, resulting in persistent oxidative stress. Instead of full regeneration, these conditions promote apoptotic signaling (through caspase-3), which leads to ongoing DNA damage, cell death, and modification. Fibrosis (such as collagen deposition) is only partially reversed, while biochemical indicators of liver damage (ALT, AST, and GGT) show some improvement. Although there is not much data on adhesion molecules (ICAM-1, VCAM-1) or acute-phase proteins (CRP) in PZQ-treated models, the general conclusion indicates that PZQ by itself is effective against the parasite but does not completely restore the immunological, oxidative, apoptotic, and fibrotic dysregulation brought on by *S. mansoni*.

The G4, G5, G6, and G7 groups observed significant decrease in contents of IL-4, IL-5, IL-6, IL-13, and TGF-β, significantly increase in levels of IL-10. The IgE and IgG1 levels were significantly reduced, while the IgG2 level was elevated. There was significant rise in significant increase in GSH content, SOD, and CAT activities with a significant reduction in MDA and NO contents. CRP, VCAM-1, and ICAM-1 levels were found to have significantly decreased. It showed a significant rise in Bcl-2 content but a significant decrease in P53, Bax, Cytochrome C, Caspase-9, Caspase-8, and Caspase-3 levels. It observed a significant reduction in Tail length, Tail moment, and Tail DNA%. Its ALT, AST, ALP, and ɤ-GT activities, and bilirubin content were significantly lower, while a notable increase in albumin content was observed compared with G2. The G7 was the best-treated group by nano-plants, recovery of liver tissue, and hepatic function tests, unlike the G4, which was less affected, ameliorated, and recovered. The synergistic effects of Fig and Olive extracts were approved previously by Reda et al. (2016); El-Morsy et al. (2022) and El-Attar et al. (2024).

There is limited and partial data on the effects of Fig and Olive on many of the specific markers in *S. mansoni*-infected mice.

Reda et al. (2016); Rezagholizadeh et al*.* (2022); El-Morsy et al. (2022) and El-Attar et al. (2024) explained that the parasite induces a Th2-mediated immune response (IL-4, IL-13, TGF-β) and oxidative stress (↑MDA, ↓GSH/SOD/CAT), which drive liver granuloma formation, regression of DNA degeneration process, cell apoptosis (↑P53, Bax, Cytochrome C, Caspases; ↓Bcl-2), and liver injury (elevated ALT, AST, ALP, GGT, bilirubin; reduced albumin). The phytochemicals Fig are Quercetin, rutin, and gallic acid, in addition to Olive, which contains oleuropein and hydroxytyrosol extracts (especially in nanoparticle form) mitigate these effects by boosting antioxidant defenses (restoring GSH, SOD, CAT), reducing inflammation (lower IL-6, CRP, VCAM-1, ICAM-1), suppressing apoptosis, and partially normalizing liver function tests, thereby reducing parasite-induced hepatic damage.

Elnahas et al. (2021) and El-Shabasy et al. (2022) supported the previous reasons by demonstrating that, during the mature worm stage, increased hepatic vascular permeability and Kupffer cell activity facilitate nanoparticle uptake, leading to a greater local effect. IL-4/IL-13-driven granulomatous and fibrotic pathways dominate. Olive and Fig polyphenols inhibit TGF-β1, α-SMA, and collagen I, reducing fibrosis. This restores bile flow (↓ALP, ↓GGT) and improves hepatocyte protein synthesis (↑albumin).

The potential of fig and olive extracts formed into nanoparticles as innovative therapeutic agents against *Schistosoma mansoni* has been underlined by recent studies. By improving the stability and bioavailability of bioactive phytochemicals, including flavonoids, phenolics, and saponins, nanoparticle encapsulation enables targeted delivery to affected tissues and continuous release. In contrast to fig-derived nanoparticles, which caused tegumental disruption and oxidative stress inside the parasite, which hampered its survival, experimental tests in mouse models showed that olive leaf extract nanoparticles drastically decreased worm load and egg deposition. Additionally, these nanoparticles enhanced the host’s antioxidant defense systems, reducing intestinal and hepatic damage brought on by schistosomiasis. Given their dual function of protecting host tissue and having direct schistosomicidal effects, fig and olive nanoparticles appear to be a viable nanotherapeutic approach for the management of schistosomiasis (El-Feky et al. 2021 and El-Sayed et al. 2022).

After *S. mansoni* infection, granuloma formation, alternative macrophage activation, and IgE/IgG1 antibody responses are supported by the strong Th2-mediated immune polarization caused by egg deposition in the liver, which also results in the up-regulation of IL-4, IL-5, IL-10, and IL-13. IL-10’s negative control aids in reducing excessive inflammation. Later on, TGF-β is also increased, which helps control tissue remodeling and fibrosis (von Bülow et al. 2022; Ismail et al. 2024).

Hepatocytes undergo apoptotic pathways as a result of this oxidative environment and chronic inflammation: pro-apoptotic P53 and Bax expression increase while anti-apoptotic Bcl-2 expression decreases, resulting in cytochrome C release from the mitochondria, activation of caspase-9, 8, and ultimately caspase-3, which causes cell death (Chen et al. 2025).

Hepatocyte damage and reduced synthetic function are reflected in the rise of liver enzymes (ALT, AST, ALP, GGT) in serum, the potential increase of total bilirubin production, and the potential decrease of albumin secretion (Mostafa et al. 2025). 

The general order is as follows: Worm infection → Th2 activation + antibody production → oxidative stress (↑NO, ROS; ↓antioxidants) → apoptotic signaling in hepatocytes → liver injury (enzyme leakage, DNA damage, fibrosis, cell death).

## Conclusion

The previous thesis done under the supervision of Dr. Eman A El-Shabasy on *Schistosoma mansoni-*infected mice and treated with Fig or Olive extracts or nanoparticles injections, finalized by using both in nano formulations to prove that, firstly, they observed synergistic effects with each other, secondly, from this study, the treatment was more curative than protective as showing the recovery of liver as assessed in cytokine profile and antibody production, oxidative stress (regressed) and antioxidants, inflammatory (decreased), apoptotic (stopped), antiapoptotic markers, liver function markers, and DNA investigations. Interestingly, it showed a return in the biomarkers to the normal healthy state.

## Supplementary Information

Below is the link to the electronic supplementary material.

Supplementary Material 1

## Data Availability

The datasets generated and analyzed during the current study are available in the file; its name is the supplementary file.
