# Supplementary material for: Immunological, anti-inflammatory, and anti-oxidant effects of Fig and Olive leaves extracts nanoparticles against Schistosoma mansoni in C57BL/6 mice
Source: Inflammopharmacology. 2026 May 20;34(7):4909–28. doi: 10.1007/s10787-026-02243-0 (PMC13391687; doi:10.1007/s10787-026-02243-0)
Supplement: Supplementary file 1 — Supplementary Material 1 [file 10787_2026_2243_MOESM1_ESM.docx]

**Table (4): Cytokine profile in liver of control and different treated mice groups**

| Mice groups  Parameters | | G1 | G2 | G3 | G4 | G5 | G6 | G7 |
| --- | --- | --- | --- | --- | --- | --- | --- | --- |
| IL-4 level  (pg/mg) | | **27^a^**  **±6.4** | **58^b^**  **±12** | **40^c^**  **±9.4** | **38^ac^**  **±4.9** | **35^ac^**  **±4.4** | **31^ac^**  **±4.9** | **29^ac^**  **±4.4** |
| %of change | ***** |  | **114.81** | **48.15** | **40.74** | **29.63** | **14.81** | **7.41** |
|  | ****** |  |  | **48.15** | **-34.48** | **-39.66** | **-46.55** | **-50.00** |
| IL-5 level  (pg/mg) | | **29^a^**  **±7** | **57^b^**  **±15** | **43^bc^**  **±8.6** | **41^c^**  **±5.6** | **38^c^**  **±4.6** | **34^c^**  **±4** | **31^c^**  **±6.2** |
| %of change | ***** |  | **96.55** | **48.28** | **41.38** | **31.03** | **17.24** | **6.90** |
|  | ****** |  |  | **-24.56** | **-28.07** | **-33.33** | **-40.35** | **-45.61** |
| IL-6 level  (pg/mg) | | **2.8^a^**  **±0.36** | **5.8^b^**  **±0.2** | **4.6^c^**  **±0.39** | **4.5^cd^**  **±0.51** | **4^cd^**  **±0.16** | **3.9^cd^**  **±0.7** | **3.6^ad^**  **±0.97** |
| %of change | ***** |  | **107.14** | **64.29** | **60.71** | **42.86** | **39.29** | **28.57** |
|  | ****** |  |  | **-20.69** | **-22.41** | **-31.03** | **-32.76** | **-37.93** |
| IL-10 level  (pg/mg) | | **101^a^**  **±11.4** | **54.9^b^**  **±14.2** | **73^c^**  **±5.69** | **78.2^cd^**  **±5.71** | **80.7^cd^**  **±3.25** | **85^ac^**  **±12.2** | **94^ad^ ±11.9** |
| %of change | ***** |  | **-45.64** | **-27.72** | **-22.57** | **-20.10** | **-15.84** | **-6.93** |
|  | ****** |  |  | **32.97** | **42.44** | **46.99** | **54.83** | **71.22** |
| IL-13 level  (pg/mg) | | **59.7^a^**  **±9.16** | **93^b^**  **±5.44** | **77.3^c^**  **±6.19** | **73^c^**  **±7.64** | **70.3^ac^**  **±7.26** | **68.8^ac^**  **±4.58** | **65.5^ac^**  **±6.66** |
| %of change | ***** |  | **55.78** | **29.48** | **22.28** | **17.76** | **15.24** | **9.72** |
|  | ****** |  |  | **-16.88** | **-21.51** | **-24.41** | **-26.02** | **-29.57** |
| TGF-β level  (pg/mg) | | **8.5^a^**  **±0.78** | **24^b^**  **±2.3** | **19^c^**  **±0.99** | **16^c^ ±1.8** | **16^d^**  **±2.2** | **13^d^**  **±1.8** | **12^d^**  **±3.1** |
| % of change | ***** |  | **182.35** | **123.53** | **88.24** | **64.71** | **52.94** | **41.18** |
|  | ****** |  |  | **-20.83** | **-33.33** | **-41.67** | **-45.83** | **-50.00** |

**G1: Negative control group (Uninfected-untreated group).**

**G2: Positive control group (Infected-untreated group).**

**G3: Infected treated with Praziquantel.**

**G4: Infected, administered with Fig-nano then Olive-nano (leaves) on the day of infection (During Cercariae stage).**

**G5: Infected, administered with Fig-nano then Olive-nano (leaves) on the 4^th^ day of infection (During Schistosomulae stage).**

**G6: Infected, administered with Fig-nano then Olive-nano (leaves) on the 11^th^ day of infection (During the Immature worm stage).**

**G7: Infected, administered with Fig-nano then Olive-nano (leaves) on the first day in week 6 of infection (During the Mature worm stage).**

**Results are presented as means ± Standard Deviation (SD)**

**and % of change (n=6 for each group).**

**(*) % of change related to G1. (**) % of change related to G2.**

**Similar letters (non-significant), Different letters (significant).**

**(a, b, c, d) indicated the significant change at *P˂*0.05.**

**Table (5): Antibody production in** **liver of control and different treated mice group**

| Mice groups  Parameters | | G1 | G2 | G3 | G4 | G5 | G6 | G7 |
| --- | --- | --- | --- | --- | --- | --- | --- | --- |
| IgE level  (ng/mg) | | **45.2^a^**  **±10.2** | **143^b^**  **±5.19** | **122^bc^**  **±12.4** | **116^c^**  **±10.3** | **111^c^**  **±14.8** | **104^c^**  **±20.5** | **97^c^**  **±21.3** |
| %of change | ***** |  | **216.37** | **169.91** | **156.64** | **145.58** | **130.09** | **114.60** |
|  | ****** |  |  | **-14.69** | **-18.88** | **-22.38** | **-27.27** | **-32.17** |
| IgG1 level  (ng/mg) | | **111^a^**  **±15.1** | **334^b^**  **±25.9** | **275^cd^**  **±6.6** | **258^cd^**  **±15.7** | **252^cd^**  **±14.6** | **239^cd^**  **±12.5** | **230^cd^**  **±21.3** |
| %of change | ***** |  | **200.90** | **147.75** | **132.43** | **127.03** | **115.32** | **107.21** |
|  | ****** |  |  | **-17.66** | **-22.75** | **-24.55** | **-28.44** | **-31.14** |
| IgG2 level  (ng/mg) | | **138^a^**  **±10.6** | **35.2^b^**  **±11.9** | **89.3^c^**  **±9.97** | **97.2^cd^**  **±10.9** | **103^cd^**  **±11.5** | **112^cd^**  **±16** | **121^ad^**  **±21.5** |
| %of change | ***** |  | **-74.49** | **-35.29** | **-29.57** | **-25.36** | **-18.84** | **-12.32** |
|  | ****** |  |  | **153.69** | **176.14** | **192.61** | **218.18** | **243.75** |

**G1: Negative control group (Uninfected-untreated group).**

**G2: Positive control group (Infected-untreated group).**

**G3: Infected treated with Praziquantel.**

**G4: Infected, administered with Fig-nano then Olive-nano (leaves) on the day of infection (During Cercariae stage).**

**G5: Infected, administered with Fig-nano then Olive-nano (leaves) on the 4^th^ day of infection (During Schistosomulae stage).**

**G6: Infected, administered with Fig-nano then Olive-nano (leaves) on the 11^th^ day of infection (During the Immature worm stage).**

**G7: Infected, administered with Fig-nano then Olive-nano (leaves) on the first day in week 6 of infection (During the Mature worm stage).**

**Results are presented as means ± Standard Deviation (SD)**

**and % of change (n=6 for each group).**

**(*) % of change related to G1. (**) % of change related to G2.**

**Similar letters (non-significant), Different letters (significant).**

**(a, b, c, d) indicated the significant change at *P˂*0.05.**

**Table (6): Oxidative stress and antioxidants in liver of control and different treated groups**

| Mice groups  Parameters | | G1 | G2 | G3 | G4 | G5 | G6 | G7 |
| --- | --- | --- | --- | --- | --- | --- | --- | --- |
| MDA content  (nmol/g) | | **774.7^a^**  **±46.88** | **1102^b^**  **±62.31** | **890^c^**  **±32.26** | **886.8^c^**  **±40.69** | **879.8^c^**  **±35.16** | **871^c^**  **±33.15** | **861.8^c^**  **±54.8** |
| %of change | ***** |  | **42.25** | **14.88** | **14.47** | **13.57** | **12.43** | **11.24** |
|  | ****** |  |  | **-19.24** | **-19.53** | **-20.16** | **-20.96** | **-21.80** |
| NO content  (µmol/g) | | **20^a^**  **±2.19** | **52.83^b^**  **±2.99** | **40.83^c^**  **±4.45** | **40.17^c^**  **±6.11** | **31.33^d^**  **±3.83** | **30.5^d^**  **±4.51** | **26.83^ad^**  **±6.80** |
| %of change | ***** |  | **164.15** | **104.15** | **100.85** | **56.65** | **52.50** | **34.15** |
|  | ****** |  |  | **-22.71** | **-23.96** | **-40.70** | **-42.27** | **-49.21** |
| GSH content  (mmol/g) | | **3.8^a^**  **±0.51** | **1.7^b^**  **±0.24** | **2.3^b^**  **±0.29** | **2.9^cd^**  **±0.2** | **3^cd^**  **±0.18** | **3.1^ad^**  **±0.54** | **3.6^ad^**  **±0.72** |
| %of change | ***** |  | **-55.26** | **-39.47** | **-23.68** | **-21.05** | **-18.42** | **-5.26** |
|  | ****** |  |  | **35.29** | **70.59** | **76.47** | **82.35** | **111.76** |
| SOD activity  (U/g) | | **197^a^**  **±12** | **126^b^**  **±7.4** | **150^c^**  **±8** | **155^c^**  **±12** | **162^c^**  **±9.4** | **164^c^**  **±7.2** | **168^c^ ±15** |
| %of change | ***** |  | **36.04** | **-23.86** | **-21.32** | **-17.77** | **-16.75** | **-14.72** |
|  | ****** |  |  | **19.05** | **23.02** | **28.57** | **30.16** | **33.33** |
| CAT activity  (U/g) | | **201^a^**  **±20** | **128^b^**  **±9.3** | **156^c^**  **±6.3** | **162^c^**  **±8.5** | **171^c^**  **±5.9** | **174^c^**  **±21** | **180^ac^**  **±22** |
| %of change | ***** |  | **-36.32** | **-22.39** | **-19.40** | **-14.93** | **-13.43** | **-10.45** |
|  | ****** |  |  | **21.88** | **26.56** | **33.59** | **35.94** | **40.63** |

**G1: Negative control group (Uninfected-untreated group).**

**G2: Positive control group (Infected-untreated group).**

**G3: Infected treated with Praziquantel.**

**G4: Infected, administered with Fig-nano then Olive-nano (leaves) on the day of infection (During Cercariae stage).**

**G5: Infected, administered with Fig-nano then Olive-nano (leaves) on the 4^th^ day of infection (During Schistosomulae stage).**

**G6: Infected, administered with Fig-nano then Olive-nano (leaves) on the 11^th^ day of infection (During the Immature worm stage).**

**G7: Infected, administered with Fig-nano then Olive-nano (leaves) on the first day in week 6 of infection (During the Mature worm stage).**

**Results are presented as means ± Standard Deviation (SD)**

**and % of change (n=6 for each group).**

**(*) % of change related to G1. (**) % of change related to G2.**

**Similar letters (non-significant), Different letters (significant).**

**(a, b, c, d) indicated the significant change at *P˂*0.05.**

**Table (7): Inflammatory markers in control and different treated groups**

| Mice groups  Parameters | | G1 | G2 | G3 | G4 | G5 | G6 | G7 |
| --- | --- | --- | --- | --- | --- | --- | --- | --- |
| Serum CRP level  (ng/ml) | | **28^a^**  **±2.9** | **96^b^**  **±11** | **70^c^**  **±6.5** | **59^c^**  **±3.8** | **44^d^**  **±8.1** | **42^d^**  **±6.8** | **39^ad^**  **±8.2** |
| %of change | ***** |  | **242.86** | **150.00** | **110.71** | **57.14** | **50.00** | **39.29** |
|  | ****** |  |  | **-27.08** | **-38.54** | **-54.17** | **-56.25** | **-59.38** |
| Hepatic VCAM-1 level  (ng/mg) | | **15^a^**  **±1** | **61^b^**  **±11** | **50^bc^**  **±6.9** | **43^cd^**  **±9.9** | **33^de^**  **±6.7** | **30^de^**  **±9** | **24^ae^**  **±5.5** |
| %of change | ***** |  | **306.67** | **233.33** | **186.67** | **120.00** | **100.00** | **60.00** |
|  | ****** |  |  | **-18.03** | **-29.51** | **-45.90** | **-50.82** | **-60.66** |
| Hepatic ICAM-1 level  (pg/mg) | | **88^a^**  **±8.6** | **204^b^**  **±13** | **156^c^**  **±4.4** | **137^cd^**  **±11** | **128^d^**  **±10** | **118^de^**  **±19** | **104^ae^**  **±15** |
| %of change | ***** |  | **131.82** | **77.27** | **55.68** | **45.45** | **34.09** | **-18.18** |
|  | ****** |  |  | **-23.53** | **-32.84** | **-37.25** | **-42.16** | **-49.02** |

**G1: Negative control group (Uninfected-untreated group).**

**G2: Positive control group (Infected-untreated group).**

**G3: Infected treated with Praziquantel.**

**G4: Infected, administered with Fig-nano then Olive-nano (leaves) on the day of infection (During Cercariae stage).**

**G5: Infected, administered with Fig-nano then Olive-nano (leaves) on the 4^th^ day of infection (During Schistosomulae stage).**

**G6: Infected, administered with Fig-nano then Olive-nano (leaves) on the 11^th^ day of infection (During the Immature worm stage).**

**G7: Infected, administered with Fig-nano then Olive-nano (leaves) on the first day in week 6 of infection (During the Mature worm stage).**

**Results are presented as means ± Standard Deviation (SD)**

**and % of change (n=6 for each group).**

**(*) % of change related to G1. (**) % of change related to G2.**

**Similar letters (non-significant), Different letters (significant).**

**(a, b, c, d) indicated the significant change at *P˂* 0.05.**

**Table (8): Apoptotic and anti-apoptotic markers in liver of control and other treated groups**

| Mice groups  Parameters | | G1 | G2 | G3 | G4 | G5 | G6 | G7 |
| --- | --- | --- | --- | --- | --- | --- | --- | --- |
| P53 level  (pg/mg) | | **146^a^**  **±12** | **286^b^**  **±14** | **239^c^**  **±13** | **216^cd^**  **±28** | **198^de^**  **±12** | **188^de^**  **±27** | **173^ae^**  **±31** |
| %of change | ***** |  | **95.89** | **63.70** | **47.95** | **35.62** | **28.77** | **18.49** |
|  | ****** |  |  | **-16.43** | **-24.48** | **-30.77** | **-34.27** | **-39.51** |
| Bax level  (pg/mg) | | **220^a^**  **±11** | **397^b^**  **±12** | **324^c^**  **±37** | **309^cd^**  **±35** | **287^d^**  **±29** | **269^ad^**  **±33** | **245^a^**  **±32** |
| %of change | ***** |  | **80.45** | **47.27** | **40.45** | **30.45** | **22.27** | **11.36** |
|  | ****** |  |  | **-18.39** | **-22.17** | **-27.71** | **-32.24** | **-38.29** |
| Bcl-2 level  (ng/mg) | | **1.1^a^**  **±0.17** | **0.38^b^**  **±0.047** | **0.63^c^**  **±0.029** | **0.7^c^**  **±0.058** | **0.79^cd^**  **±0.1** | **0.82^cd^**  **±0.15** | **0.93^d^**  **±0.14** |
| %of change | ***** |  | **-65.45** | **-42.73** | **-36.36** | **-28.18** | **-25.45** | **-15.45** |
|  | ****** |  |  | **65.79** | **84.21** | **107.89** | **115.79** | **144.74** |
| Cytochrome C level  (pg/mg) | | **154^a^**  **±4.7** | **287^b^**  **±15** | **249^c^**  **±7.9** | **204^d^**  **±23** | **188^de^**  **±19** | **182^ad^**  **±29** | **164^ae^ ±18** |
| %of change | ***** |  | **86.36** | **61.69** | **32.47** | **22.08** | **18.18** | **6.49** |
|  | ****** |  |  | **-13.24** | **-28.92** | **-34.49** | **-36.59** | **-42.86** |
| Caspase-9 level  (pg/mg) | | **346^a^**  **±49** | **998^b^**  **±67** | **835^c^**  **±35** | **772^c^**  **±33** | **743^c^**  **±52** | **736^c^**  **±57** | **589^d^**  **±89** |
| %of change | ***** |  | **188.44** | **141.33** | **123.12** | **114.74** | **112.72** | **70.23** |
|  | ****** |  |  | **-16.33** | **-22.65** | **-25.55** | **-26.25** | **-40.98** |
| Caspase-8 level  (ng/mg) | | **1.8^a^**  **±0.18** | **7.1^b^**  **±0.45** | **5.4^c^**  **±0.42** | **4.6^c^**  **±0.48** | **4.1^c^**  **±0.5** | **3.7^cd^**  **±0.53** | **3.2^d^**  **±0.73** |
| % of change | ***** |  | **294.44** | **200.00** | **155.56** | **127.78** | **105.56** | **77.78** |
|  | ****** |  |  | **-23.94** | **-35.21** | **-42.25** | **-47.89** | **-54.93** |
| Caspase-3 level  (ng/mg) | | **1.9^a^**  **±0.078** | **8.7^b^**  **±0.39** | **7.1^c^**  **±0.41** | **6.3^c^ ±0.39** | **4.4^d^**  **±1.1** | **3.5^de^**  **±0.74** | **2.7^e^**  **±0.98** |
| % of change | ***** |  | **357.89** | **273.68** | **231.58** | **131.58** | **84.21** | **42.11** |
|  | ****** |  |  | **-18.39** | **-27.59** | **-49.43** | **-59.77** | **-68.97** |

**G1: Negative control group (Uninfected-untreated group).**

**G2: Positive control group (Infected-untreated group). G3: Infected treated with Praziquantel.**

**G4: Infected, administered with Fig-nano then Olive-nano (leaves) on the day of infection (During Cercariae stage).**

**G5: Infected, administered with Fig-nano then Olive-nano (leaves) on the 4^th^ day of infection (During Schistosomulae stage).**

**G6: Infected, administered with Fig-nano then Olive-nano (leaves) on the 11^th^ day of infection (During the Immature worm stage).**

**G7: Infected, administered with Fig-nano then Olive-nano (leaves) on the first day in week 6 of infection (During the Mature worm stage).**

**Results are presented as means ± Standard Deviation (SD). and % of change (n=6 for each group). (*) % of change related to G1. (**) % of change related to G2.**

**Similar letters (non-significant), Different letters (significant).**

**(a, b, c, d) indicated the significant change at *P˂*0.05.**

**Table (9): Liver function tests in serum of control and different treated mice groups**

| Mice groups  Parameters | | G1 | G2 | G3 | G4 | G5 | G6 | G7 |
| --- | --- | --- | --- | --- | --- | --- | --- | --- |
| ALT activity  (U/L) | | **33.83^a^**  **±5.74** | **166.5^b^**  **±8.26** | **119^c^**  **±31.9** | **84.17^cd^**  **±21.88** | **74.83^d^**  **±21.88** | **73.33^d^**  **±22.2** | **66.83^ad^**  **±15.99** |
| %of change | ***** |  | **392.17** | **251.76** | **148.8** | **121.19** | **116.76** | **97.55** |
|  | ****** |  |  | **-28.53** | **-49.45** | **-55.06** | **-55.96** | **-59.86** |
| AST activity  (U/L) | | **37.5^a^**  **±5.612** | **227^b^**  **±18.86** | **119.3^c^**  **±31.39** | **108.2^cd^**  **±17.14** | **91.17^cd^**  **±23.68** | **78^d^**  **±25.15** | **73.5^d^**  **±22.77** |
| %of change | ***** |  | **505.33** | **218.13** | **188.53** | **143.12** | **108.00** | **96.00** |
|  | ****** |  |  | **-47.44** | **-52.33** | **-59.84** | **-65.64** | **-67.62** |
| ALP activity  (U/L) | | **53.5^a^**  **±16.21** | **195.5^b^**  **±36.52** | **165.3^b^**  **±7.174** | **119.8^c^**  **±36.97** | **81.17^ac^**  **±19.36** | **84.33^b^**  **±5.239** | **72.33^ac^**  **±16.03** |
| %of change | ***** |  | **265.42** | **208.97** | **123.93** | **51.72** | **42.67** | **35.20** |
|  | ****** |  |  | **-15.45** | **-38.72** | **-58.48** | **-60.96** | **-63.00** |
| ɤ-GT activity  (U/L) | | **29.33^a^**  **±4.131** | **65.33^b^**  **±12.01** | **55.5^bc^**  **±13.03** | **49.5^bd^**  **±12.37** | **40.83^acd^**  **±5.492** | **37.33^ad^**  **±7.815** | **33.5^ad^ ±7.092** |
| %of change | ***** |  | **122.74** | **89.23** | **68.77** | **39.21** | **27.28** | **14.22** |
|  | ****** |  |  | **-15.05** | **-24.23** | **-37.50** | **-42.86** | **-48.72** |
| Albumin content  (g/dl) | | **3.8^a^**  **±0.59** | **1.4^b^**  **±0.25** | **2.3^c^**  **±0.27** | **2.4^c^**  **±0.35** | **2.6^c^**  **±0.15** | **2.9^ac^**  **±0.29** | **3.1^ac^**  **±0.91** |
| %of change | ***** |  | **-63.16** | **-39.47** | **-36.84** | **-31.58** | **-23.68** | **-18.42** |
|  | ****** |  |  | **64.29** | **71.43** | **85.71** | **107.14** | **121.43** |
| Bilirubin content  (mg/dl) | | **0.59^a^**  **±0.25** | **1.9^b^**  **±0.3** | **1.2^c^**  **±0.29** | **1.1^c^ ±0.17** | **0.91^ac^**  **±0.25** | **0.78^ac^**  **±0.22** | **0.62^a^**  **±0.38** |
| % of change | ***** |  | **222.03** | **103.39** | **86.44** | **54.24** | **32.20** | **5.08** |
|  | ****** |  |  | **-36.84** | **-42.11** | **-52.11** | **-58.95** | **-67.37** |

**G1: Negative control group (Uninfected-untreated group).**

**G2: Positive control group (Infected-untreated group).**

**G3: Infected treated with Praziquantel.**

**G4: Infected, administered with Fig-nano then Olive-nano (leaves) on the day of infection (During Cercariae stage).**

**G5: Infected, administered with Fig-nano then Olive-nano (leaves) on the 4^th^ day of infection (During Schistosomulae stage).**

**G6: Infected, administered with Fig-nano then Olive-nano (leaves) on the 11^th^ day of infection (During the Immature worm stage).**

**G7: Infected, administered with Fig-nano then Olive-nano (leaves) on the first day in week 6 of infection (During the Mature worm stage).**

**Results are presented as means ± Standard Deviation (SD)**

**and % of change (n=6 for each group).**

**(*) % of change related to G1. (**) % of change related to G2.**

**Similar letters (non-significant), Different letters (significant).**

**(a, b, c, d) indicated the significant change at *P˂*0.05.**

**Table (10): DNA damage assessments in the hepatocytes of control and different treated mice groups**

| Mice groups  Parameters | | G1 | G2 | G3 | G4 | G5 | G6 | G7 |
| --- | --- | --- | --- | --- | --- | --- | --- | --- |
| Tail length  (µm) | | **0.88^a^**  **±0.066** | **3.65^b^**  **±0.144** | **3.22^c^**  **±0.105** | **3.21^c^**  **±0.192** | **3.16^c^**  **±0.0833** | **2.94^d^**  **±0.119** | **2.89^d^**  **±0.111** |
| %of change | ***** |  | **314.77** | **265.91** | **264.77** | **259.09** | **234.09** | **228.41** |
|  | ****** |  |  | **-11.78** | **-12.05** | **-13.42** | **-19.45** | **-20.82** |
| Tail moment  (UNIT) | | **0.59^a^**  **±0.0629** | **12.9^b^**  **±0.807** | **10.3^c^**  **±0.447** | **10.2^c^**  **±0.785** | **9.65^c^**  **±0.389** | **8.47^d^**  **±0.498** | **7.92^d^**  **±0.516** |
| %of change | ***** |  | **2086.44** | **1645.76** | **1628.81** | **1535.59** | **1335.59** | **1242.37** |
|  | ****** |  |  | **-20.16** | **-20.93** | **-25.19** | **-34.34** | **-38.60** |
| Tail DNA  (%) | | **0.67^a^**  **±0.0613** | **3.54^b^**  **±0.128** | **3.19^cd^**  **±0.078** | **3.18^cd^**  **±0.1** | **3.05^d^**  **±0.118** | **2.88^de^**  **±0.102** | **2.74^e^**  **±0.121** |
| %of change | ***** |  | **428.36** | **376.12** | **374.63** | **355.22** | **329.85** | **308.96** |
|  | ****** |  |  | **-9.89** | **-10.17** | **-13.84** | **-18.64** | **-22.60** |

**G1: Negative control group (Uninfected-untreated group).**

**G2: Positive control group (Infected-untreated group).**

**G3: Infected treated with Praziquantel.**

**G4: Infected, administered with Fig-nano then Olive-nano (leaves) on the day of infection (During Cercariae stage).**

**G5: Infected, administered with Fig-nano then Olive-nano (leaves) on the 4^th^ day of infection (During Schistosomulae stage).**

**G6: Infected, administered with Fig-nano then Olive-nano (leaves) on the 11^th^ day of infection (During the Immature worm stage).**

**G7: Infected, administered with Fig-nano then Olive-nano (leaves) on the first day in week 6 of infection (During the Mature worm stage).**

**Results are presented as means ± Standard Deviation (SD)**

**and % of change (n=6 for each group).**

**(*) % of change related to G1. (**) % of change related to G2.**

**Similar letters (non-significant), Different letters (significant).**

**(a, b, c, d) indicated the significant change at *P˂* 0.05.**
